# New section in journal of translational medicine: patient-targeted molecular therapies

**DOI:** 10.1186/1479-5876-10-92

**Published:** 2012-05-15

**Authors:** Adrian Bot, Francesco Chiappelli

**Affiliations:** 1 Chief Scientific Officer, Kite Pharma, Inc., 10924 Le Conte Avenue, Los Angeles, CA, 90024, USA; 2 UCLA School of Dentistry, AAAS Fellow, Fulbright Specialist, CHS 63-090, 10833 Le Conte Avenue, Los Angeles, CA, 90095-1668, USA; 3 Evidence-Based Decisions Practice-Based Research Network (EBD-PBRN.org), California, USA

## Abstract

This Editorial announces a new section in the Journal of Translational Medicine: Patient-Targeted Molecular Therapies. This section is dedicated to the dissemination of targeted molecular therapies in context of patient-centered outcomes research and evidence-based clinical decisions. The focus on patient-targeted molecular therapies – spanning small molecules and biomolecules alike – stems from the unprecedented growth in this arena. This is consonant with the overall objective of the Journal of Translational Medicine, which seeks out to expand firmly to other vast areas of medicine in the domain of translational science, viewed here as the transaction between translational research and translational effectiveness. As we inaugurate this new section in Journal of Translational Medicine, with its mission described in detail in this Editorial, we invite interested scientists to submit their work for publication.

## 

Analyses of current limitations and deficiencies in health care in the context of the Affordable care Act, signed into law by President Obama in March 2010 [1], indicate that national and global trends for the next decades will emphasize patient-centered outcomes research of molecular-targeted evidence-based interventions. Treatment modalities in medicine, nursing and dentistry will be increasingly called upon, and designed to coalesce translational research - going from the patient to the laboratory bench and back to the patient (NIH definition) - with translational effectiveness - integrating the best available evidence for optimizing evidence-based health care interventions in specific clinical settings (AHRQ definition).

There has been unparalleled and continuous progress in research and development in broad areas of molecular medicine resulting in novel platform technologies, drugs, biomolecules and companion diagnostics on the market and in development. Additionally, post-approval testing of novel medicines or therapeutic combinatorial modalities with the aim to improve the standard of care has been a steady continuous source of information with impact to the healthcare. Key to these advances is the dynamism of this process, and the magnitude and complexity of resulting informational output, warranting iterative application of appropriate research and evaluation tools across entire domains of medicine, to identify and promote standard of care or outline state of the art.

Therefore, innovative models of evidence-based patient-centered molecular-targeted interventions are timely and critical. Evidence-based medicine/dentistry/nursing, through the process of comparative efficacy and effectiveness research and review for clinical practice (CEERAP) pursues the identification of the best available evidence gained from the scientific method to clinical decision-making [2,3]. CEERAP seeks to assess the strength of evidence in terms both of what works in a clinical setting (efficacy), and of the risks, costs and benefits of treatments and diagnostics (effectiveness). Methodologically, CEERAP involves research synthesis across all the available published studies and “gray” literature scientific sources that pertain to the specific clinical or diagnostic question defined by the patient (P), the interventions (I) under consideration (C) for the clinical outcome (O) sought, often within the constraints of a given interval of time (T) and clinical setting (S) (hence, the acronym PICO-TS). CEERAP is a systematic process of research that follows the hypothesis-driven scientific process, and that yields specialized research reports, which are termed systematic reviews. The data generated in a systematic review are analyzed statistically by various means that include acceptable sampling analysis and meta-analysis. Not all systematic reviews are so structured as to incorporate a meta-analysis, and not all meta-analysis reports are set up and organized as a systematic PICO-TS research. Systematic reviews and meta-analysis reports in the context of CEERAP converge to seeking a consensus of the best available evidence for clinical relevance. Calls for dissemination of this novel approach to health care are also increasingly pressing.

In response to this urgency, the Journal of Translational Medicine now inaugurates a new section, entitled *Patient-Targeted Molecular Therapies*, which is dedicated to the dissemination of targeted molecular therapies in the context of patient-centered outcomes research and evidence-based clinical decisions. Therefore, the focus on patient-targeted molecular therapies – spanning small molecules and biomolecules alike – stems from the unprecedented growth in this arena, fueled by scientific progress in understanding the molecular pathogenesis of disease.

The section *Patient-Targeted Molecular Therapies* provides avenues for the discussion of translational effectiveness pertaining to diverse therapeutic areas including inflammation, degenerative disorders, cancer, infection, metabolism, neurologic, cardiovascular to genetic disorders. The section considers pathologies that pertain to the micro-environment and local immunoregulation and epigenetics, as well as systems immunophysiology. The section addresses chronic and acute immune-related pathologies, from autoimmune disease, to HIV/AIDS, from immune deficiencies to osteo-immunopathology, from inborn errors of immune metabolism to psycho-neuroendocrine-immune pathologies. This section also considers non-immunological disorders, and conditions that are at the interface with immunopathology with both facets: the drug (or therapeutic intervention) and the companion biomarkers.

The purpose of this new section is consonant with the overall objective of the Journal of Translational Medicine, which – with traditional and diligent interest in immune therapies - seeks out to expand firmly to other vast areas of medicine. This section on *Patient-Targeted Molecular Therapies* will be complemented by an interest in prognostic and predictive biomarkers, as well as companion diagnostics as they are a critical tool in the assessment or application of molecular targeted therapeutics, and emphasize patient-targeted research for personalized medicine, now subsumed under the terminology “patient-centered outcomes research”. As molecular targeted therapies – through an increased potential for clinical benefit and data-rich clinical design - carry the benefit of yielding informative data sets in individual patients during smaller trials in early development, a focus will be to outline emerging evidence of efficacy or toxicity for innovative drug candidates.

In brief, as the informational output regarding more mature or approved molecular targeted therapies evolves across multiple development programs and studies, the specific content of this section will include research products in the form of systematic reviews, clinically relevant complex systematic reviews and meta-analyses. It will also include reviews of the best available evidence, in the form of critical evidence reviews and evidence-based policies. The scientific focus of this section on *Patient-Targeted Molecular Therapies* in Journal of Translational Medicine specifically addresses the best available evidence about the translational effectiveness of biomarkers-guided targeted molecular therapies of established medical and dental clinical conditions.

With this opening editorial, we announce and inaugurate this new section in Journal of Translational Medicine. We invite interested scientists to submit their work for publication.
